# Health and wellness coaching positively impacts individuals with chronic pain and pain-related interference

**DOI:** 10.1371/journal.pone.0236734

**Published:** 2020-07-27

**Authors:** Zachary D. Rethorn, Robert W. Pettitt, Emily Dykstra, Cherie D. Pettitt

**Affiliations:** 1 Doctor of Physical Therapy Division, Duke University, Durham, North Carolina, United States of America; 2 Rocky Mountain University of Health Professions, Provo, Utah, United States of America; 3 WGU Academy, Western Governors University, Salt Lake City, Utah, United States of America; Monash University, AUSTRALIA

## Abstract

**Objectives:**

Health and wellness coaching (HWC) interventions have been reported to improve health outcomes for individuals with chronic diseases such as diabetes, cardiovascular disease, or cancer. However, HWC also holds potential as an effective intervention within a biopsychosocial chronic pain management framework. The aim of the present study was to evaluate the effects of HWC on individuals with chronic pain.

**Methods:**

Participants were referred by their primary care provider or insurance company to a comprehensive telephonic 12-month pain management HWC program. Relationships between pain outcomes and physical and psychological factors were retrospectively analyzed. Mixed linear-effects modeling explored whether physical and psychological variables were associated with pain outcomes over time.

**Results:**

Four hundred nineteen participants (female, 58.9%; mean age, 54.8) enrolled in the program and 181 completed the intervention. After 12 months in the program, statistically and clinically significant reductions were observed for pain intensity (Hedges’ g = 1.00) and pain-related interference (Hedges’ g = 1.13). Linear mixed-effects modeling indicated that improvements in physical functioning and psychological factors were associated with improvements in pain intensity.

**Discussion:**

Our results provide a novel analysis on the effects of HWC on chronic pain and pain-related interference. HWC appears to be a promising intervention to improve pain-related outcomes in a population with chronic pain. Further investigation of HWC as an intervention for chronic pain is warranted.

## Introduction

Chronic pain is defined by the International Association for the Study of Pain as “persistent or recurrent pain lasting longer than 3 months [1].” In addition, chronic pain causes functional and structural changes to the nervous system that result in continued ongoing pain separate from the initial cause. As such, experts agree it becomes its own separate medical condition [2] and will include separate codes in the 11^th^ revision of the International Classification of Diseases [3]. Between 30–40% of U.S. adults have chronic pain, exceeding the number of Americans living with diabetes, heart disease, or cancer [4]. The Institute of Medicine (IOM) estimates chronic pain cost the United States between $560 and $635 billion annually in direct medical treatment costs and lost productivity [5]. Therefore, effective chronic pain treatment has become a moral imperative [3, 6].

Chronic pain is complex and affects biological, psychological, and social dimensions making it a biopsychosocial condition that requires interdisciplinary approaches for treatment and management [7]. Multiple reports including the IOM consensus report, the Centers for Disease Control and Prevention Guideline for Prescribing Opioids for Chronic Pain [8], and the Department of Health and Human Services’ National Pain Strategy [9] have called for an expanded view of treating chronic pain to include biological, psychological, and social factors. The IOM consensus report shifts the initial focus of trying to find and resolve the cause of chronic pain to improving the experience of individuals living with chronic pain by enhancing functioning and quality of life [6, 8, 9]. As a result, exercise therapy, cognitive behavioral therapy, and non-opioid medications are promoted as first-line treatments for chronic pain.

The shifted focus on the biopsychosocial aspects of chronic pain necessitates an interdisciplinary team of practitioners. Often these interdisciplinary teams include primary care providers, psychologists, pharmacists, and physical therapists [10]. However, the emergence of the health & wellness coaching (HWC) may offer added value to the interdisciplinary care team by helping the patient identify their personal values and goals to determine the most effective pain management plan recognizing that anxiety, depression, stress, insomnia, and disability are closely associated with long-term pain [11–14].

HWC holds great potential for advancing healthy behavior change and stemming the rising tide in prevalence of chronic disease [15]. The National Board for Health & Wellness Coaching (NBHWC) describes health and wellness coaching as partnering

with clients seeking self-directed, lasting changes, aligned with their values, which promote health and wellness and, thereby, enhance well-being. In the course of their work health and wellness coaches display unconditional positive regard for their clients and a belief in their capacity for change, and honoring that each client is an expert on his or her life, while ensuring that all interactions are respectful and non-judgmental [16].

To date, health coaching research demonstrates positive effects on health outcomes for participants with various chronic diseases such as diabetes, cardiovascular disease, and cancers [15, 17–19]. Effective coaching interventions have been delivered by a variety of health professionals including nurses, nutritionists, exercise physiologists, physical therapists, and psychologists [15, 17, 20–22].

Health behavior change is the driving force of a health and wellness coach’s role which aligns clearly with helping individuals struggling with a variety of biopsychosocial factors related to their health conditions. Thus, the purpose of this study was to determine the effectiveness of a novel HWC-based biopsychosocial program for treating individuals with chronic pain. The present retrospective observational study had three goals. The first was to determine if health and wellness coaching for chronic pain was associated with improvements in clinical pain outcomes including pain intensity and pain-related interference. The second goal was to examine if psychological factors were improved following the program. The third goal was to explore if physical and psychological factors were associated with clinical pain outcomes over time. We hypothesized that we would observe improvements in pain intensity, pain-related interference, and psychological factors following the program.

## Materials and methods

### Reporting guidelines

This study followed the Strengthening the Reporting of Observational Studies in Epidemiology (STROBE) Statement [23]. The STROBE statement was created to benchmark reporting of observational studies to improve the transparency of the research process.

### Study design

This study was a retrospective, nonrandomized analysis of consecutive participants who were enrolled between Jan 1, 2010 and December 31, 2018 in Take Courage Coaching (Bozeman, MT). Baseline patient and clinical data were extracted from the program. Study procedures were approved by the Rocky Mountain University of Health Professions Institutional Review Board.

### Clinical program

Beginning in 2010, an expert in health and wellness coaching (HWC) designed a 12-month comprehensive telephonic HWC program which integrates pain education, self-care skills training, goal-setting guidance, self-monitoring tools, social support, and career guidance for individuals with nonmalignant persistent pain. The program’s mission was to help people who are experiencing debilitating pain return to productive, rewarding lives and has been further described elsewhere [24]. Participants were referred from a medical provider or their insurer and were administered the Pain Outcomes Questionnaire (POQ) [25] prior to beginning the program, 6-months after beginning the program, and 12 months after beginning the program.

Participants committed to 30-minute individual coaching sessions once per week for 52 weeks and 60-minute group coaching sessions once per week for 52 weeks. The individual sessions aimed to facilitate the client finding motivation to begin managing pain and improving their quality of life. Participants were also asked their motivation and confidence related to pain-management behaviors and set SMART goals [26] each week. Goals were patient-derived and not prescribed by the coaches. The group sessions were solution-focused with a curriculum of 52 different lessons including: mind-body connection, neuroplasticity, medication, self-compassion, strengths and values, and mindfulness. The end goal of the coaching was for the patient to become motivated to make positive changes in their overall state of wellness. The program culminated with a client-designed wellness-based maintenance plan.

All coaches completed a 75-hour training program that included topics related to neuroplasticity, coaching modalities such as Motivational Interviewing, Appreciative Inquiry, and Strengths and Values-Based coaching, along with information regarding the psychology and physiology of pain and best practices in pain management. The primary communication style used by the coaches was motivational interviewing [27]. All coaches were required to participate in mentored calls, pass a written exam, and subjected to a recorded call evaluation process using the Motivational Interviewing Treatment Integrity (MITI) Scale to ensure coaching performance fidelity and reliability [28]. After the first year, the coaches were assessed with the MITI scale every 6 months for their second year and then once per year thereafter. In addition, all coaches engaged in a formal peer mentoring process. There were no minimal requirements for the HWCs hired during the time period of data collection. Most were previous graduates of the pain management program. All coaches were hired and trained by Take Courage Coaching and this company currently only uses National Board Certified Health and Wellness Coaches.

### Patient measures

#### Demographics and clinical data

Participants self-reported their clinical and demographic information at baseline and 12 months. Information included gender, race, years of education completed, presence of a disability claim, employment status, duration of pain, and identified pain sites. Using a numeric rating scale (0–10) participants reported their average pain over the last week and their acceptable level of pain. Reporting average pain using NRS has been previously utilized in populations with persistent pain [29]. The acceptable level of pain refers to a level of pain which a patient would be comfortable rather than acceptance of the pain condition. Thus, it can be thought of as a measure of participants’ expectations regarding treatment.

#### Pain outcome measure

The POQ is a 19 item multidomain pain treatment outcome instrument administered at baseline, 6 months, and at 12 months [25]. Pain-related functioning is measured in five domains: mobility (4 items), activities of daily living (4 items), negative affect (5 items), vitality (3 items), and fear of activity (2 items). Each domain was assessed through an 11-point Likert scale (0 = “never” to 10 = “always”). Items within each domain are summed and a total score is aggregated. Higher scores indicate greater pain-related impairment. The POQ is a reliable and valid measurement of chronic pain treatment outcomes with good internal consistency for all subscales (α = 0.78 to 0.90) with the exception of the fear subscale (α = .59), which is composed of only two items [25]. The POQ also demonstrates moderate to strong convergent and discriminant validity (*r* ≥ 0.30) and thus delineates distinct aspects of the chronic pain experience [25].

#### Treatment satisfaction

At the end of the 12-month HWC program, participants were asked five questions from the POQ which reflect treatment satisfaction. Each question asks the patient to rate their satisfaction with treatment on a scale from 0 to 10 (0 = “no satisfaction” and 10 = “complete satisfaction). The questions assess overall treatment, staff (personality and competence), treatment schedule, and whether they would recommend the treatment to others.

### Data analysis

Statistical analyses were performed according to the intention-to-treat principle where all participants were included in the primary analyses. Linear mixed-effects analyses with *lme4* [30] were performed to determine differences in baseline, 6 month, and 12 month scores for each outcome variable as well as for exploratory analyses of the relationships between pain intensity and 1) the physical functioning POQ domains related to mobility and activities of daily living, and 2) the psychological functioning POQ domains related to vitality, negative affect, and fear. We chose to use linear mixed-effect modeling because it is flexible in handling missing repeated measures outcome data and robust in resolving non-independence between variables [31, 32]. Sensitivity analyses were performed to determine whether the associations varied when excluding participants who did not complete the HWC program.

For the physical functioning model, we included mobility and activities of daily living as predictors and pain intensity as the outcome. As fixed effects we included intercepts for time, mobility, activities of daily living with interaction terms for time. As random effects we included intercepts for participants and timepoints as well as by-subject and by-timepoint random slopes for the effects of mobility and activities of daily living with an unstructured variance-covariance structure and restricted maximum likelihood estimation [33].

For the psychological functioning model, we included vitality, negative affect, and fear as predictors and pain intensity as the outcome. As fixed effects we included intercepts for time, vitality, negative affect, and fear with interaction terms for time. As random effects we included intercepts for participants and timepoints as well as by-subject and by-timepoint random slopes for the effects of vitality, negative affect, and fear with an unstructured variance-covariance structure and restricted maximum likelihood estimation [33].

For all models, residual plots were inspected for obvious deviations from homoscedasticity or normality. P-values were obtained by likelihood ratio tests of the full model with the effect in question against the model without the effect in question. Differences in the baseline characteristics between participants who completed the HWC program and those who did not complete the HWC program were evaluated using *t* tests for continuous variables and chi-square tests for categorical variables. Internal consistency of the POQ was evaluated using Cronbach’s alpha. For all analyses, *p* < 0.05 was considered significant. Analyses were completed using IBM SPSS Statistics Version 25 (IBM, Chicago, IL) and R version 4.0.0 (R Foundation for Statistical Computing, Vienna, Austria).

## Results

### Sample characteristics

**Fig 1:**
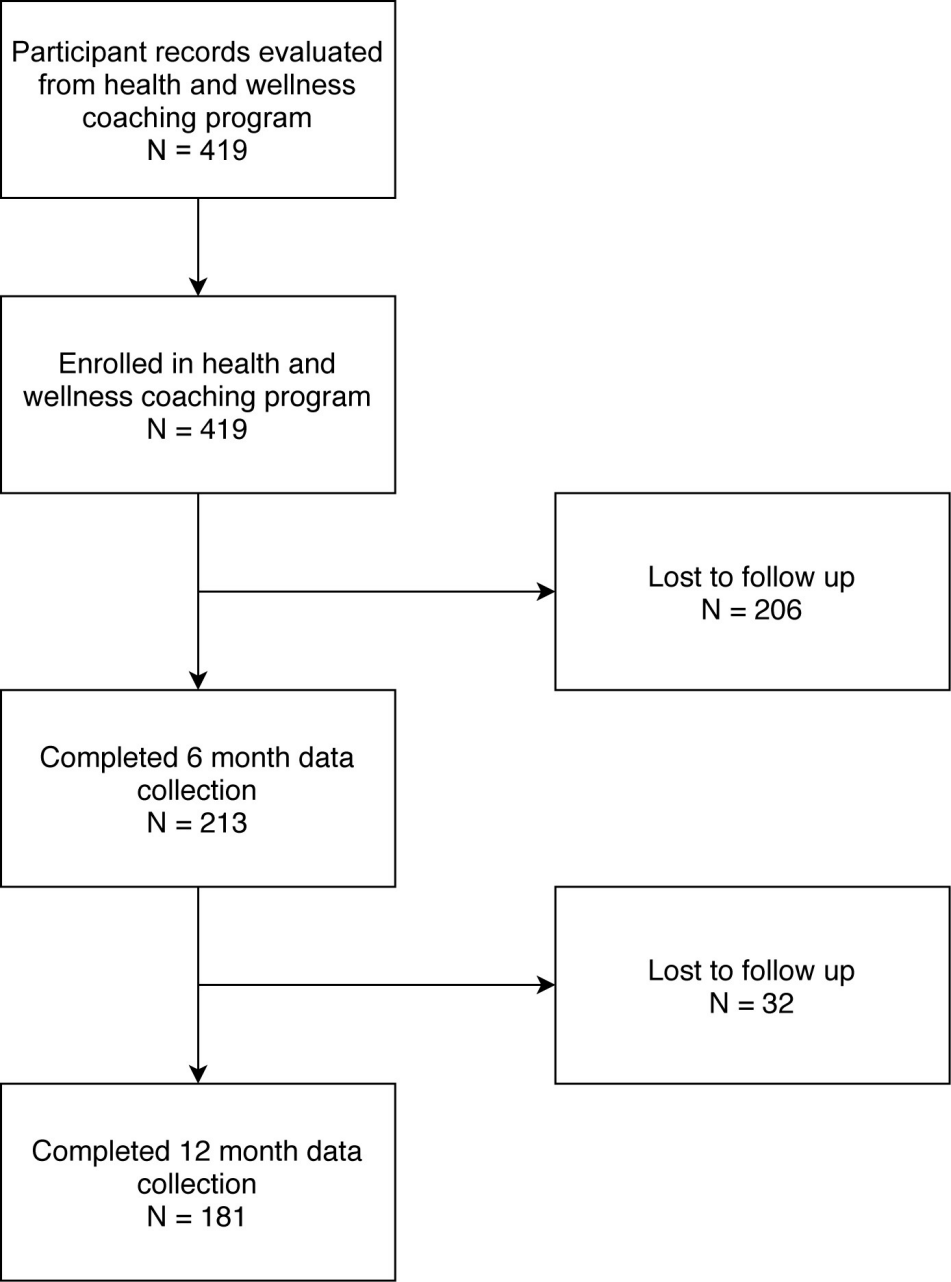
Flow diagram of participants through study.

In total, 419 participants enrolled in the program and completed baseline data collection. Two hundred thirteen had 6-month and 181 had 12-month outcomes data that allowed for retrospective analysis (see Fig 1 for more details). Individuals included in this sample were recruited from 43 states in the United States and were primarily female (58.9%) and Caucasian (83.8%). The mean age of participants was 54.8 (SD = 12.5). Participant characteristics and demographics are presented in Table 1. The average pain duration and number of pain sites reported were 10.4 (SD = 10.7) years and 6.9 (SD = 4.4) sites, respectively. The average attendance rate for participants who completed the program was 92% compared to 75% average attendance rate for those who did not complete the program.

**Table 1:** Sample characteristics and demographics.

	Completed HWC program (n = 181)	Partially completed HWC program (n = 238)	*P* value
Demographic			
Mean Age (SD)	54.1 (10.7)	56.0 (11.8)	.36
Female gender, *n* (%)	113 (62.4)	134 (56.3)	.12
Caucasian race, *n* (%)	155 (90.6)	196 (83.1)	**.008**
Mean years of education (SD)	15.4 (8.0)	13.9 (3.3)	**.015**
Disability claim filed, *n* (%)	46 (28.2)	65 (27.7)	.87
Employment, *n* (%)			**.003**
Retired	23 (12.7)	41 (14.5)	
Unemployed	111 (61.3)	153 (54.1)	
Part-time employed	18 (9.9)	14 (4.9)	
Full-time employed	20 (11.0)	30 (10.6)	
Pain			
Mean pain duration in years (SD)	9.8 (9.1)	10.8 (11.5)	.95
Mean number of pain sites (SD)	6.9 (4.4)	6.9 (4.4)	.90
Mean acceptable pain (SD)	3.5 (1.7)	3.5 (1.63)	.21
Low back pain interferes most, *n* (%)	52 (28.7)	81 (28.6)	.31
Location of pain, *n* (%)			.57
Abdomen	44 (24.3)	54 (19.1)	
Arm/hand	90 (49.7)	116 (41.0)	
Buttocks	67 (37.0)	90 (31.8)	
Chest	32 (17.7)	44 (15.5)	
Face	32 (17.7)	36 (12.7)	
Fingers	58 (32.0)	88 (31.1)	
Foot	88 (48.6)	118 (41.7)	
Genitals	19 (10.5)	24 (8.5)	
Head	74 (40.9)	94 (33.2)	
Jaw	32 (17.7)	36 (12.7)	
Leg	117 (64.6)	156 (55.1)	
Low back	131 (72.4)	186 (65.7)	
Mid back	82 (45.3)	114 (40.3)	
Neck	103 (56.9)	130 (45.9)	
Shoulder	105 (58.0)	125 (44.2)	
Toes	57 (31.5)	86 (30.4)	
Upper back	79 (43.6)	94 (33.2)	
Other	38 (21.0)	49 (16.3)	

### Comparison of baseline, 6 month, and 12 month outcomes

**Fig 2:**
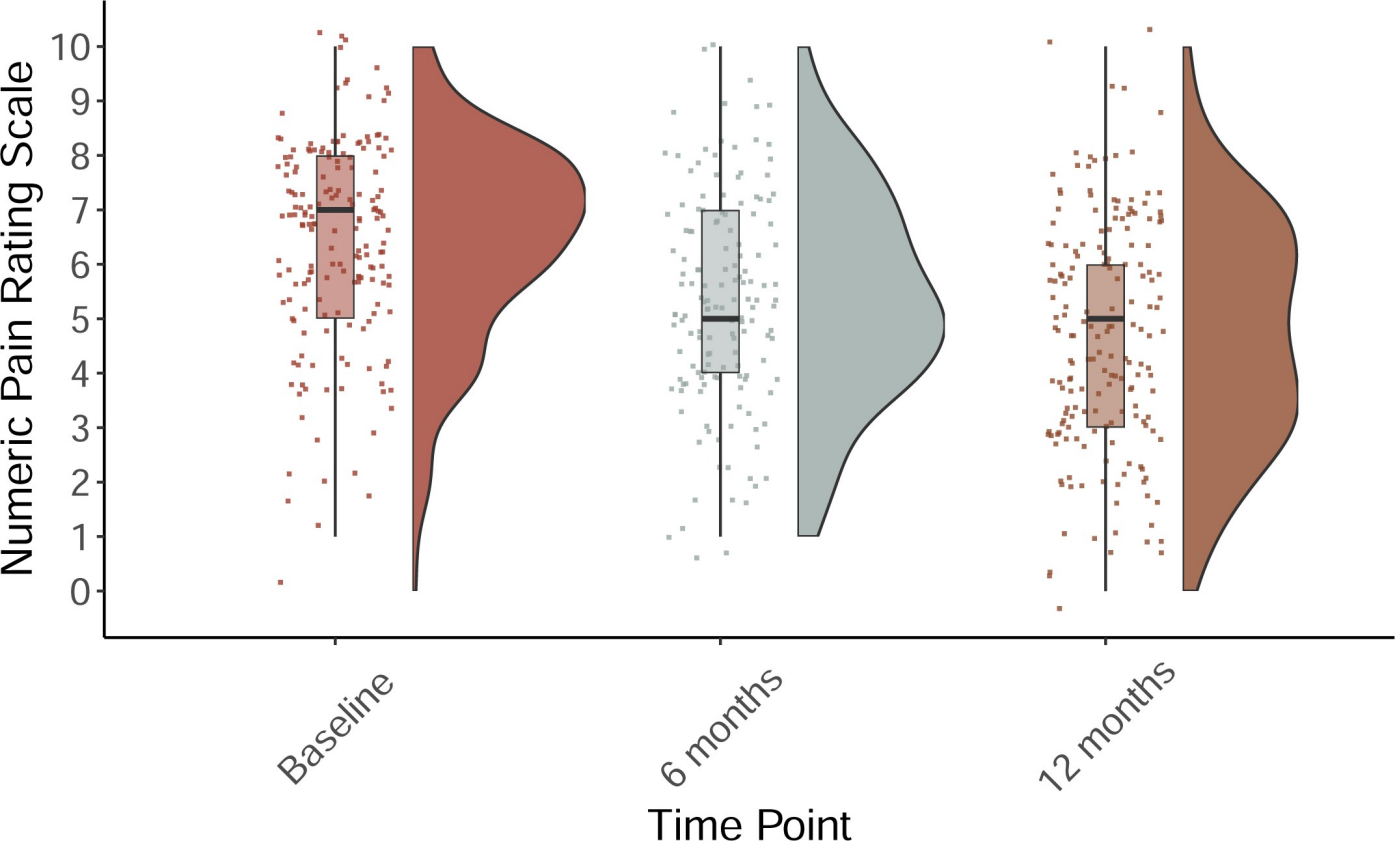
Comparison of the Pain Outcomes Questionnaire (POQ) total scores by timepoint.

**Fig 3:**
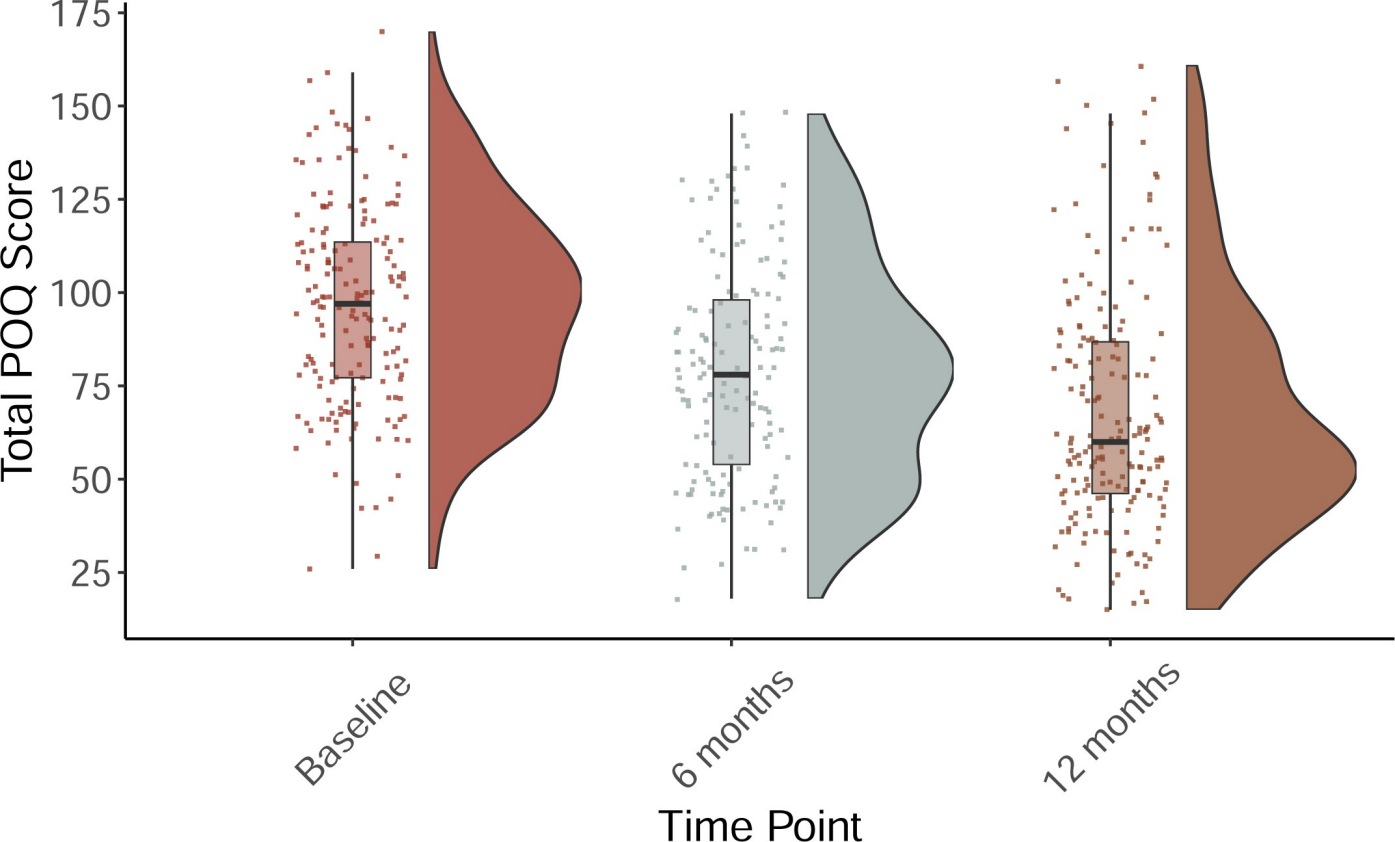
Comparison of the pain numeric rating scale by timepoint.

We used the total POQ score as a global index of pain-related functioning. Internal consistency of the POQ was good at baseline (α = 0.73) and at 12 months (α = 0.75). Linear mixed effects models were used to examine the differences in baseline, 6 month, and 12 month outcomes are presented in Table 2. Pre- and post-treatment POQ and pain intensity ratings are presented in Figs 2 and 3. Results indicate statistically significant improvements in all variable scores except fear with large effect sizes (g > 0.8) for pain intensity, mobility, vitality, negative affect, and composite pain-related functioning. A small effect size (g <0.5) was observed for pain-related impairment in completing ADLs as well as in pain-related fear and avoidance.

**Table 2:** POQ domain scores by timepoint.

	Baseline		6 month		12 month			
POQ Scale	Mean*	SD	Mean*	SD	Mean*	SD	*p*	Effect size (g)^†^
Pain intensity	6.68	1.77	5.41	2.03	4.71	2.12	< 0.001	1.00
Mobility	22.45	10.72	17.52	12.03	13.64	11.29	< 0.001	0.81
ADL	11.10	10.48	8.79	9.75	6.59	9.27	< 0.001	0.44
Vitality	19.78	5.18	16.08	5.54	13.85	5.87	< 0.001	1.10
Negative affect	28.41	11.03	21.61	11.49	17.93	11.26	< 0.001	0.94
Fear	11.23	3.81	11.61	3.52	11.00	2.25	0.236	0.07
Total POQ score	101.50	28.88	82.08	32.61	67.74	31.89	< 0.001	1.13

* Positive values indicate reductions in POQ scale scores.

^†^ Effect sizes are reported using Hedges’ *g*.

POQ = Pain Outcomes Questionnaire; ADL = Activities of Daily Living; SD = standard deviation.

### Exploratory linear mixed-effects regression analyses

Exploratory linear mixed-effects regression analyses using pain intensity as a dependent variable and psychological factors and physical factors as predictor variables are reported in Tables 3 and 4. Pain intensity was associated with psychological (marginal R^2^ = .297) and physical functioning variables (marginal R^2^ = .335). Pain-related vitality, negative affect and mobility were most consistently associated with positive reductions in pain intensity. Sensitivity analyses utilizing only participants who completed the HWC coaching revealed similar associations for psychological (marginal R^2^ = .264) and physical functioning variables (marginal R^2^ = .322) (S1 Appendix). Multicollinearity was assessed using variance inflation factor which was less than 3 for all predictors. Visual inspection of residual plots did not reveal any obvious deviations from homoscedasticity or normality.

**Table 3:** Linear mixed-effects analysis of psychological factors associated with pain intensity.

	Pain			
*Predictors*	*Estimates*	*CI*	*p*	*df*
(Intercept)	5.16	4.34 – 5.98	**<0.001**	789.00
6 month timepoint	-1.45	-2.66 – -0.25	**0.018**	789.00
12 month timepoint	-3.30	-4.56 – -2.05	**<0.001**	789.00
Baseline timepoint: vitality	0.06	0.02 – 0.09	**0.001**	789.00
6 month timepoint: vitality	0.04	0.00 – 0.09	**0.045**	789.00
12 month timepoint: vitality	0.07	0.03 – 0.11	**0.002**	789.00
Baseline timepoint: negative affect	0.03	0.02 – 0.05	**<0.001**	789.00
6 month timepoint: negative affect	0.07	0.05 – 0.09	**<0.001**	789.00
12 month timepoint: negative affect	0.06	0.04 – 0.08	**<0.001**	789.00
Baseline timepoint: fear	-0.05	-0.09 – -0.01	**0.018**	789.00
6 month timepoint: fear	-0.04	-0.10 – 0.02	0.204	789.00
12 month timepoint: fear	0.08	0.01 – 0.15	**0.020**	789.00
ICC	0.46			
N _participant_	416			
Observations	803			
Marginal R^2^ / Conditional R^2^	0.297 / 0.618			

CI = confidence interval, df = degrees of freedom, ICC = intraclass correlation coefficient, N = number.

**Table 4:** Linear mixed-effects analysis of physical functioning factors predicting pain intensity.

	Pain intensity			
*Predictors*	*Estimates*	*CI*	*p*	*df*
(Intercept)	5.31	4.94 – 5.68	**<0.001**	793.00
6 month timepoint	-1.27	-1.73 – -0.80	**<0.001**	793.00
12 month timepoint	-1.78	-2.24 – -1.32	**<0.001**	793.00
Baseline timepoint: mobility	0.05	0.03 – 0.06	**<0.001**	793.00
6 month timepoint: mobility	0.06	0.03 – 0.08	**<0.001**	793.00
12 month timepoint: mobility	0.08	0.06 – 0.11	**<0.001**	793.00
Baseline timepoint: ADL	0.02	0.01 – 0.04	**0.004**	793.00
6 month timepoint: ADL	0.04	0.01 – 0.07	**0.004**	793.00
12 month timepoint: ADL	0.01	-0.02 – 0.05	0.386	793.00
ICC	0.39			
N _participant_	416			
Observations	804			
Marginal R^2^ / Conditional R^2^	0.335 / 0.591			

ADL = Activities of Daily Living, CI = confidence interval, df = degrees of freedom, ICC = intraclass correlation coefficient, N = number.

### Treatment satisfaction

Participants reported high overall levels of satisfaction with the HWC program at 12 months. The mean satisfaction score at 12 months was 48.15 (SD = 3.60) and the median score was 50 out of a maximum possible score of 50.

## Discussion

We examined a sample of individuals enrolled in a HWC program with the goal of exploring: (1) if HWC for chronic pain was associated with meaningful reduction in self-reported pain, including pain intensity and pain-related interference, (2) if psychological factors improved during the intervention, and (3) if improvements in physical and psychological factors were associated with pain intensity. First, we observed that HWC was associated with clinically meaningful reductions in pain intensity and pain-related interference at 6 months and 12 months. Second, health and wellness coaching was associated with improved psychological pain-related functioning and physical functioning related to pain. Third, psychological and physical pain-related functioning were associated with pain intensity over time.

The present findings highlight the potential clinical utility of HWC to improve pain intensity as well as psychological and physical pain-related functioning. To our knowledge, this is the first HWC program designed to assist individuals with chronic pain to improve pain-related functioning. Interestingly, pain-related fear improved only minimally compared to other psychological constructs. We believe that result may partially be explained by the low reliability observed by the fear scale with Cronbach α of 0.55 and 0.65 at pre- and post-treatment, respectively. Alternatively, the relatively modest fear levels demonstrated at baseline may be due to the persistence of the pain experience among individuals in this sample.

In the present sample negative affect, vitality, and mobility were most consistently associated with pain intensity. It is interesting to note that the individuals enrolled in the HWC program, which was not designed to directly address pain intensity, nevertheless noted improved pain intensity over time. Perhaps changes in mobility led to improved physical activity–one behavior which has been shown to improve chronic pain [34]. More work will be required to provide a comprehensive understanding of the mechanisms underlying HWC for chronic pain in order to improve treatment for this population.

While extensive reviews include a comprehensive overview of treatment approaches for chronic pain management [35], we are limiting our comparisons with those in the literature most similar to HWC. Our findings are encouraging and have important clinical implications for chronic pain management when compared with other alternative therapies. For instance, one meta-analytic review concluded that the utilization of acceptance-based interventions had an effect size of 0.37 for pain reduction [36]; whereas, cognitive behavioral therapy interventions produce reduction of pain on the order of ~0.50 [37]. A recent online intervention observed that the inclusion of biopsychosocial elements in comparison to usual evoke small to moderate improved outcomes [38]. The present findings compare favorably with an effect size of 1.13. Future research should confirm these findings with more robust research designs and follow up periods as nonrandomized studies often overestimate effect size compared to more rigorous designs.

Some limitations should be considered when interpreting the efficacy of HWC in the present study. HWC is designed to honor the patient’s autonomy and encourages them to explore alternative ways to manage their pain. However, only 43% of the participants completed the HWC program. This dropout rate is higher than other HWC programs designed to improve health behaviors related to chronic diseases,[15] but is similar to other yearlong behavioral interventions among patients with chronic pain [39]. Patient expectations regarding treatment for chronic pain are increasingly recognized as an important factor to address. Patients generally have high expectations regarding pain reduction following an intervention [40]. Because the HWC program was not directly designed to reduce pain, but instead aimed to improve quality of life, participant expectations may have influenced the dropout rate.

Observed improvements in POQ scores should be interpreted in light of alternative interventions that were unaccounted for such as physical therapy interventions and pharmacological therapy the participants in our study received concurrently with the HWC program. Variances in usual clinical care between participants may have impacted program results. Spontaneous recovery may explain some improvements though published data suggest that incidence of spontaneous recovery from persistent pain is low [41]. Our study population may not be representative of other chronic pain populations. Our sample had, on average, longer pain durations, more pain sites, and were more likely to be unemployed compared to samples used for normative data in chronic pain [42, 43]. These factors may have influenced our results and decreased the generalizability of them to other populations. Further, all data collected were self-reported, and results are subject to possible over- or under-reporting. Future work should include objective measures of functioning. Despite these limitations, these initial results are promising and demonstrate the need for further evaluations of HWC as an intervention for individuals with chronic pain.

HWC delivered through digital means may become more important as more healthcare shifts online. Initial research suggests that HWC through videoconferencing may be as effective as in-person HWC to promote weight loss [44–46]. Telephone-based coaching has been found to be as effective as in-person coaching for weight loss and weight maintenance [47–49]. Beyond the advantages of digital delivery of HWC related to reduced transmission of infectious diseases, digital interventions may be especially important for those who are in isolated or rural communities. However, more research is needed to directly compare the efficacy of in-person and coaching delivered through digital media. Clarifying which populations may benefit the most from a digital service delivery pathway is also needed.

In summary, HWC may serve as an effective therapy to chronic pain management programs and trained health and wellness coaches may play an important role as part of the multidisciplinary healthcare team working within a biopsychosocial framework for chronic pain management. In addition, telephonic HWC interventions provide a means of treatment that may help those who are unable to travel, need additional support after completing intensive treatment, or as a follow-up to revisit strategies and skills for relapse prevention. Evaluation of HWC for individuals with chronic pain demonstrated that participants overall exhibited positive gains in pain intensity as well as aspects of physical and psychological health. These findings among this sample are promising and are a call to action to conduct more robust research including randomized clinical trials in the future.

## Supporting information

S1 Appendix Linear mixed-effects sensitivity analyses excluding individuals who were lost to follow up. (DOCX)

S1 File STROBE checklist. (DOCX)

S2 File Anonymized data set. (XLSX)
