# Supplementary material for: Health and wellness coaching positively impacts individuals with chronic pain and pain-related interference
Source: PLoS One. 2020 Jul 27;15(7):e0236734. doi: 10.1371/journal.pone.0236734 (PMC7384647; doi:10.1371/journal.pone.0236734)
Supplement: S1 Appendix — (DOCX) [file pone.0236734.s001.docx]

**S1 Appendix. Linear mixed-effects sensitivity analyses excluding individuals who were lost to follow up.**

**S1 Table 1. Sensitivity analysis excluding individuals who were lost to follow up of physical functioning factors predicting pain intensity.**

|  | **Pain intensity** | | | |
| --- | --- | --- | --- | --- |
| *Predictors* | *Estimates* | *CI* | *p* | *df* |
| (Intercept) | 4.92 | 4.38 – 5.47 | **<0.001** | 492.00 |
| 6 month timepoint | -0.83 | -1.42 – -0.24 | **0.006** | 492.00 |
| 12 month timepoint | -1.45 | -2.00 – -0.89 | **<0.001** | 492.00 |
| Baseline timepoint : mobility | 0.06 | 0.03 – 0.09 | **<0.001** | 492.00 |
| 6 month timepoint : mobility | 0.06 | 0.03 – 0.08 | **<0.001** | 492.00 |
| 12 month timepoint: mobility | 0.08 | 0.06 – 0.11 | **<0.001** | 492.00 |
| Baseline timepoint : ADL | 0.02 | -0.00 – 0.05 | 0.096 | 492.00 |
| 6 month timepoint : ADL | 0.04 | 0.00 – 0.07 | **0.036** | 492.00 |
| 12 month timepoint: ADL | 0.01 | -0.02 – 0.05 | 0.443 | 492.00 |
| ICC | 0.42 | | | |
| N _participant_ | 179 | | | |
| Observations | 503 | | | |
| Marginal R^2^ / Conditional R^2^ | 0.322 / 0.607 | | | |

ADL = Activities of Daily Living, CI = confidence interval, df = degrees of freedom, ICC = intraclass correlation coefficient, N = number

**S1 Table 2. Sensitivity analysis excluding individuals lost to follow up of psychological factors predicting pain intensity.**

|  | **Pain intensity** | | | |
| --- | --- | --- | --- | --- |
| *Predictors* | *Estimates* | *CI* | *p* | *df* |
| (Intercept) | 4.77 | 3.57 – 5.98 | **<0.001** | 489.00 |
| 6 month timepoint | -0.45 | -2.04 – 1.13 | 0.574 | 489.00 |
| 12 month timepoint | -2.94 | -4.42 – -1.46 | **<0.001** | 489.00 |
| Baseline timepoint : vitality | 0.04 | -0.00 – 0.09 | 0.069 | 489.00 |
| 6 month timepoint : vitality | 0.02 | -0.02 – 0.07 | 0.326 | 489.00 |
| 12 month timepoint: vitality | 0.07 | 0.02 – 0.11 | **0.004** | 489.00 |
| Baseline timepoint : negative affect | 0.04 | 0.01 – 0.06 | **0.001** | 489.00 |
| 6 month timepoint : negative affect | 0.07 | 0.04 – 0.09 | **<0.001** | 489.00 |
| 12 month timepoint: negative affect | 0.06 | 0.04 – 0.08 | **<0.001** | 489.00 |
| Baseline timepoint : fear | -0.02 | -0.08 – 0.04 | 0.433 | 489.00 |
| 6 month timepoint : fear | -0.07 | -0.14 – 0.00 | 0.056 | 489.00 |
| 12 month timepoint: fear | 0.08 | 0.01 – 0.15 | **0.018** | 489.00 |
| ICC | 0.51 | | | |
| N _participant_ | 179 | | | |
| Observations | 503 | | | |
| Marginal R^2^ / Conditional R^2^ | 0.264 / 0.640 | | | |

CI = confidence interval, df = degrees of freedom, ICC = intraclass correlation coefficient, N = number
